# DEVELOPMENT OF A SCALE TO MEASURE CONTACT WITH OLDER ADULTS

**DOI:** 10.1093/geroni/igz038.315

**Published:** 2019-11-08

**Authors:** Robert C Intrieri, Maria Kurth

**Affiliations:** 1 Western Illinois University, Macomb, Illinois, United States; 2 OSU Oregon State University, Corvallis, Oregon, United States

## Abstract

Allport (1954) hypothesized that intergroup contact would reduce prejudice that an in-group member would experience toward an out-group member. Allport held that positive effects of intergroup contact would occur when four conditions were met: (a) equal group status within the situation, (b) common goals, (c) intergroup cooperation, and (d) the support of authorities, law, or custom. Although contact with older adults is an important influence on attitudes toward older people, no psychometrically adequate measures of contact exist. Specifically, this study examined the factor structure of an instrument to measure contact with older adults. The convenience sample consisted of 188 women and 282 men (n = 470). Mean ages for men and women were 21.06 (SD = 2.28) and 20.88 (SD = 3.09), respectively (Mtotal = 20.99, SDtotal = 2.63). Participants were predominantly Caucasian (n=295, 62.6%), African American (n=67, 14.2%), Hispanic/Latino/a (n=63, 13.4%), and other minorities comprising the remaining 9.8%. Results of a confirmatory factor analysis showed the three factor model exhibited a reasonable fit to the data X2 (41, N = 471) = 281.81; p<.0001, CFI =.954; TLI =.938; RMSEA =. 000 (90% CI, 0.100-0.124) SRMS = .054. Results and further adjustments to the model will be discussed.

